# A Systematic Literature Review and Indirect Treatment Comparison of Efficacy of Repository Corticotropin Injection versus Synthetic Adrenocorticotropic Hormone for Infantile Spasms

**DOI:** 10.36469/jheor.2021.18727

**Published:** 2021-01-27

**Authors:** Michael S. Duchowny, Ishveen Chopra, John Niewoehner, George J. Wan, Beth Devine

**Affiliations:** 1 Nicklaus Children’s Hospital, Miami, FL; 2 Manticore Consultancy, Bethesda, Maryland, United States; 3 Mallinckrodt Pharmaceuticals, Bedminster, New Jersey, United States; 4 University of Washington, Seattle, Washington, United States

**Keywords:** adrenocorticotropic hormone, indirect treatment comparison, infantile spasms, meta-analysis, repository corticotropin injection, systematic literature review

## Abstract

**Background:** Infantile spasms is a rare disease characterized by distinct seizures and hypsarrhythmia. Adrenocorticotropic hormone (ACTH) is available as a natural product (repository corticotropin injection, [RCI]; Acthar® Gel) and as synthetic analogs. RCI is a naturally-sourced complex mixture of purified ACTH analogs and other pituitary peptides approved by the United States Food and Drug Administration as a monotherapy for the treatment of infantile spasms. RCI is commonly used in the United States. Outside the United States, synthetic analogs of ACTH—synthetic ACTH1-24 (tetracosactide) and synthetic ACTH1-39 (corticotropin carboxymethyl-cellulose [CCMC])—are used. The efficacy of RCI may differ from that of synthetic ACTH treatments based on the structure of peptide; however, no head-to-head clinical trials have compared the efficacy of RCI and synthetic ACTH treatments.

**Objective:** A systematic review and indirect treatment comparison of clinical trials was conducted to assess the comparative efficacy of RCI and synthetic ACTH treatments in infantile spasms.

**Methods:** A search was conducted in MEDLINE, EMBASE, and Cochrane databases through September 30, 2020. Relevant clinical trials on RCI or synthetic ACTH therapy and reporting either cessation of spasms or resolution of hypsarrhythmia, separately or as a combined outcome were included. A Bayesian indirect treatment comparison using a fixed-effects model was used for comparative efficacy.

**Results:** Of 473 citations screened, 21 studies were reviewed qualitatively. In the indirect treatment comparison of six eligible clinical trial studies, the odds of achieving efficacy outcomes were five to eight times greater with RCI than with tetracosactide and 14 to 16 times greater than CCMC. This translated to a risk reduction of 10% to 14% and 40% to 50% with RCI versus tetracosactide and CCMC, respectively. For every two to five patients treated, RCI improved efficacy outcomes in one additional patient compared to synthetic ACTH (adjusted number needed-to-treat).

**Conclusions:** Based on the available limited evidence, results suggest RCI may be more efficacious for infantile spasms than synthetic ACTH treatments. Our findings provide a blueprint to inform the design of future prospective studies for the treatment of infantile spasms.

## INTRODUCTION

Infantile spasms is a rare disorder with an estimated global incidence of two to three per 10 000 live births and a lifetime prevalence of 2 to 3.5 per 10 000 live births.^1,2^ Infantile spasms are characterized by distinct seizures and a representative electroencephalographic (EEG) pattern known as hypsarrhythmia.^1,2^ The onset of seizures typically occurs between four to eight months after birth.^3^ Infantile spasms involve sudden, rapid contractions of the trunk and limbs of varied intensity that last one to two seconds and may result in a brief loss of consciousness.^3,4^ Although the spasms resolve with time, many children have long-term complications, including severe epilepsy and severe psychomotor retardation, which places a considerable burden on the patients and their caregivers.^5^

Early diagnosis and timely treatment of infantile spasms can reduce epileptic symptoms and associated complications.^6^ A delay in treatment is associated with poor developmental outcomes in the long term.^2^ Further, short-duration treatment with improved efficacy is preferred to avoid major side effects with treatments for infantile spasms.^2^ Current treatment options include hormonal treatments, vigabatrin, and oral corticosteroids.^2,7^ Hormonal treatments, such as intramuscular adrenocorticotropic hormone (ACTH) have been used since the 1950s.^6^ ACTH is used in the short-term to achieve cessation of spasms and/or resolution of hypsarrhythmia.^2^ ACTH is recommended as the first-line treatment for controlling spasms in infants.^2^ Treatment is initiated with a maximum dose of ACTH over 2 weeks followed by the dose tapering.^2,8^

Repository corticotropin injection (RCI) or Acthar^®^ Gel is a naturally-sourced complex mixture of purified ACTH analogs and other pituitary peptides.^8^ RCI stimulates endogenous corticosteroid production and is an agonist for all five melanocortin receptors.^8,9^ Melanocortin receptor activation by ACTH has been shown to have direct and indirect anti-inflammatory and immunomodulatory effects.^9^ RCI is approved by the United States Food and Drug Administration as a first-line treatment of infantile spasms.^8^ In addition, recent practice recommendations from the American Academy of Neurology and the Child Neurology Society concluded that RCI may be more effective in the treatment of infantile spasms than other treatment options.^10,11^ RCI is commonly used in the United States; a recent survey suggested that 84% of clinicians prescribed RCI for the treatment of infantile spasms.^12^

ACTH is available as a natural product and as a synthetic analog.^13^ Outside the United States, synthetic analogs of ACTH—synthetic ACTH_1-24_ (tetracosactide) and synthetic ACTH_1-39_ (corticotropin carboxymethyl-cellulose [CCMC])—are used.^6,14^ The efficacy of RCI may differ from that of synthetic ACTH treatments based on the structure of peptide;^15^ however, there are no head-to-head trials comparing the efficacy of RCI and synthetic ACTH treatments.

Randomized controlled trials are the gold standard for determining the efficacy of treatments, and in the absence of direct comparisons, a systematic literature review and meta-analysis can generate sufficient power from available trials to create a single, more precise estimate of treatment effect.^16^ A systematic literature review and network meta-analysis using indirect treatment comparison (ITC) were conducted to assess the comparative efficacy of RCI versus synthetic ACTH therapies in infants with infantile spasms.

## METHODS

### Data Sources and Search Strategy

A comprehensive electronic search of PubMed/MEDLINE, EMBASE, Cochrane Controlled Register of Trials and Cochrane Database of Systematic Reviews was conducted to identify published peer-reviewed studies. No restrictions were used on the publication date and the search was conducted through September 30, 2020. An additional search was conducted in relevant conference proceedings since 2009 to identify recent literature within the last 10 years. A longer time frame was allowed for a comprehensive search. The search strategy was based on the population, intervention, comparators, outcomes, and study design criteria outlined in Table 1, and the search strategy is provided in Table S1. In addition, a bibliographic search of the relevant studies and systematic literature reviews was conducted manually to ensure that all relevant studies were captured.

**Table 1.** PICOS Criteria

**Criteria**	**Description**
**Population**	Infants with infantile spasms; age: 2-24 months Infantile spasms is a rare disorder characterized by distinct seizures and a representative electroencephalographic (EEG) pattern known as hypsarrhythmia.
**Intervention**	Repository corticotropin injection
**Comparator(s)**	Two comparators were assessed separately. Synthetic ACTH_1-24_ (tetracosactide) Synthetic ACTH_1-39_ (corticotropin carboxymethyl-cellulose)
**Outcomes**	Efficacy outcomes were assessed at 2-4 weeks of follow-up. Efficacy outcomes comprised: Cessation of spasms was defined as no observed spasms based on clinical criteria, either reported by parents or trained observers. Resolution of hypsarrhythmia was evaluated based on video EEG monitoring. Paired outcome comprising both cessation of spasms and resolution of hypsarrhythmia.
**Study Design**	Controlled/trial studies

Abbreviations: ACTH, adrenocorticotropic hormone; PICOS, participants, interventions, comparisons, outcomes, and study design

### Study Selection Criteria

The study selection was based on the standard Preferred Reporting Items for Systematic Reviews and Meta-Analyses.^17^ Two independent reviewers (IC and JN) screened each title, abstract, and full-text article to identify relevant studies. Disagreements were resolved by discussion and consensus with input by an independent third reviewer (GJW), as necessary. Data were extracted by two independent reviewers (IC and JN) and stored in Microsoft^®^ Excel 2019 (Redmond, Washington, United States).

Studies included infants with infantile spasms treated with RCI or synthetic ACTH therapies compared to placebo, standard supportive care (e.g., oral corticosteroids), or other drugs (e.g., vigabatrin and nitrazepam). Further, a common comparator arm for RCI and synthetic ACTH treatment was required for studies to be included in the ITC. Studies reporting at least one of the efficacy outcomes of interest were included. Only clinical trials were included as they provide the highest level of evidence in a meta-analysis.^18^ Observational studies were excluded as they are likely to introduce bias and increase the risk of producing an imprecise effect estimate.^19^ Studies with inadequate information on patient selection and outcomes were excluded. Case reports, abstract reports, letters, editorials, surveys, studies of non-original data, and reviews were excluded. Studies for indications other than infantile spasms were also excluded.

### Efficacy Outcomes

Efficacy outcomes included the number of infants who achieved cessation of spasms and resolution of hypsarrhythmia, reported separately, and as a paired outcome at follow-up. The goals of treatment for infantile spasms should include complete cessation of spasms and resolution of hypsarrhythmia.^2^ Cessation of spasm is the most common short-term outcome evaluated by clinical trials and observational studies. Cessation of spasms was defined as no observed spasms based on clinical criteria, either reported by parents or trained observers. The resolution of hypsarrhythmia was evaluated based on video EEG monitoring. Effective cessation of spasms and resolution of hypsarrhythmia is considered as an “all‐or‐none” event rather than a graded response to treatment.^2^ Review of these trials suggested that the paired outcome was based on both clinical and EEG criteria.

### Methodologic Quality and Risk of Bias

The quality of each extracted controlled/clinical trial was assessed with the Jadad score^20^ and Cochrane risk-of-bias tool.^21^ The Jadad score evaluates trial randomization, masking, and withdrawal/dropout methods and yields an overall score ranging from 0 to 5; a higher score indicates better methodological quality.^20^ The Cochrane tool assesses the intra-study risk of bias across the following domains: sequence generation, allocation concealment, blinding of participants, blinding of outcome assessment, incomplete outcome data, selective outcome reporting, and other sources of bias.^21^ Each domain is answered with a ‘low’, ‘unclear’, or ‘high’ risk of bias.

### Statistical Analyses

Comprehensive assessments were conducted to ensure unbiased and accurate estimates. Similarity assessment was conducted across studies by qualitatively reviewing study, patient, and treatment characteristics. Consistency assessment was not performed as the analysis did not include head-to-head trials. The selection of fixed-effects or random-effects model was based on deviance information criterion (DIC), a Bayesian method for comparison of models to determine model fit. Models with a small DIC value are supported by the data as they provide the best short-term predictions.^22^ Fixed-effects model had a DIC value lower than or similar to the random-effects model for all comparisons, suggesting a better model fit and lack of heterogeneity. Further, a DIC difference between fixed-effects and random-effects models greater than 10 is considered substantial.^22^ However, there was no substantial difference between fixed-effects and random-effects model estimates. Based on model fit diagnostics and lack of heterogeneity, a fixed-effects model was selected for the analyses. The fixed-effects model is also preferred due to the small number of clinical trials included in this ITC.^23^

A Bayesian framework for the ITC was used to generate estimates of relative treatment outcomes to account for any heterogeneity in the estimates. The Bayesian approach relies on the estimates from the probability distribution function from the observed data.^24^ This approach combines data from all clinical trials into a statistically integrated analysis to generate a pooled estimate of each intervention’s relative treatment effect compared to all others.^25^ The Bayesian approach is apt for conducting network meta-analysis with a small number of trials and limited sample size.^24^ The Bayesian approach also accounts for any unobservable heterogeneity in the estimates. The Markov chain Monte Carlo method was applied to the fixed-effects model until convergence was achieved. The Bayesian model framework used the following parameters: number of chains = 3, number of turning iterations = 10 000, number of simulation iterations = 100 000, thinning interval = 10, number of inference samples = 10 000, and variance scaling factor = 2.5. Models were programmed and executed using WinBUGS version 1.4.3 (Cambridge, United Kingdom).^26^

Treatment effects for efficacy outcomes are estimated as odds ratios (ORs) of RCI compared to synthetic ACTH. Uncertainty around point estimates is provided as a 95% credible interval (CrI), which indicates that the outcome estimates fall within the given range with 95% probability. A CrI not including one is considered statistically significant. In addition, adjusted absolute risk reduction and the adjusted number needed to treat were also calculated. The number needed to treat was defined as improved efficacy outcomes in one additional patient treated with RCI versus tetracosactide or CCMC.

## RESULTS

### Study Selection and Characteristics

**Figure 1.**
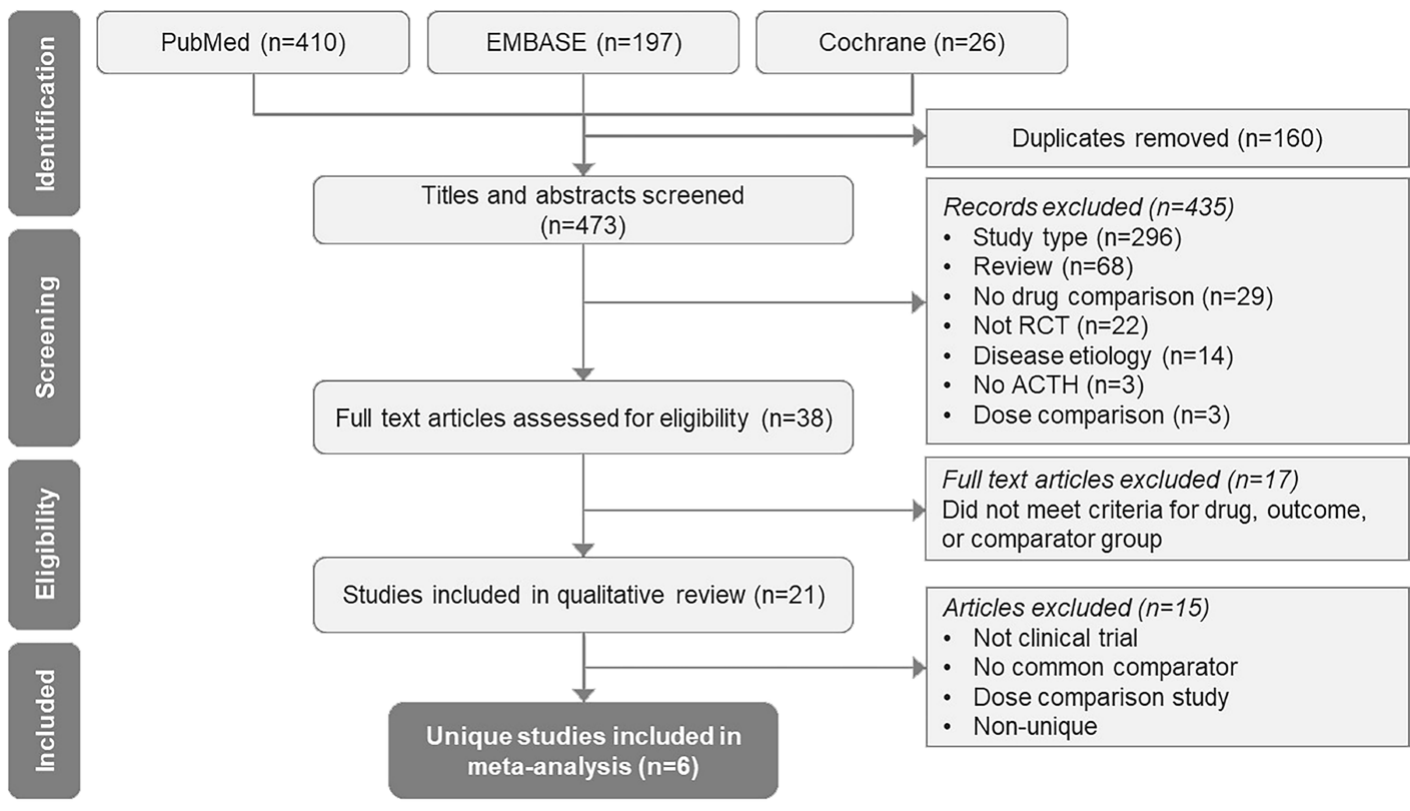
PRISMA Flow Diagram for the Systematic Literature Review (executed September 30, 2020) Abbreviations: ACTH, adrenocorticotropic hormone; PRISMA, Preferred Reporting Items for Systematic Reviews and Meta-Analyses; RCT, randomized controlled trial

A total of 473 citations were identified initially, out of which 38 full-text articles were retained after excluding studies based on the pre-specified study selection criteria. Out of the 38 citations screened; 21 citations were reviewed qualitatively and six clinical trials were included in the ITC based on the selection criteria (Figure 1).^6,14,27–30^ The characteristics of studies excluded from ITC and reason for exclusion are provided in Table S2.

**Figure 2.**
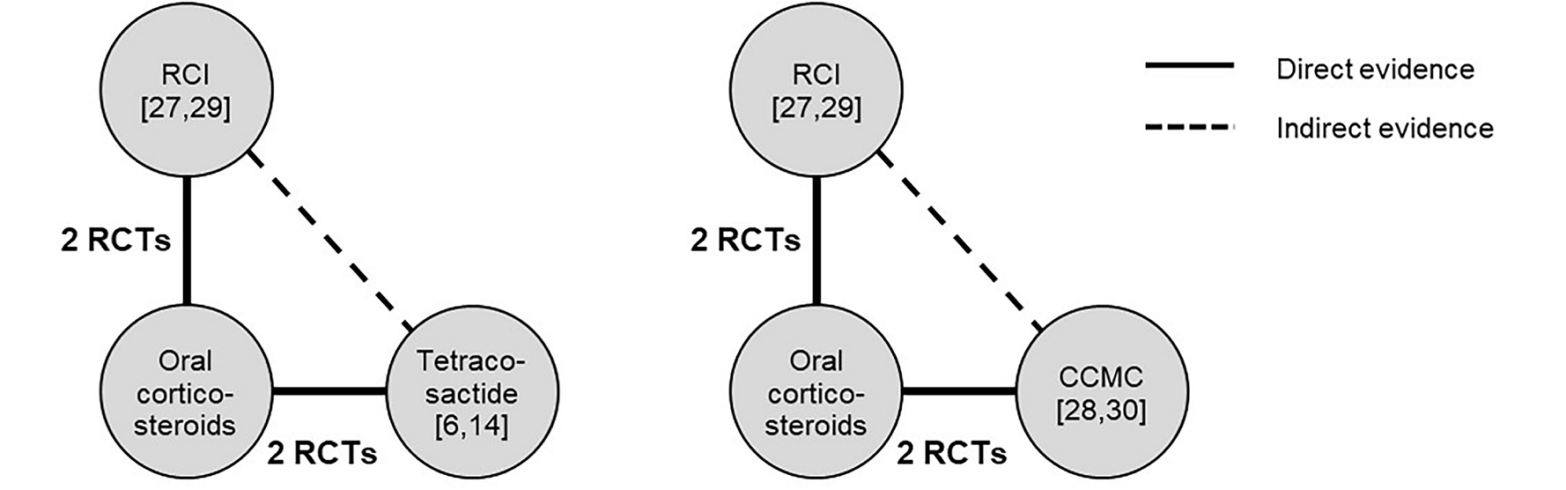
Network Plots for the RCI Versus Synthetic ACTH Comparisons Abbreviations: ACTH, adrenocorticotropic hormone; CCMC, corticotropin carboxymethyl-cellulose; OCS, oral corticosteroids; RCI, repository corticotropin injection; RCT, randomized controlled trial The treatment effect of RCI evaluated by abstracting the treatment effect of the common arm, using the general equation: *d_RCI/ACTH_ = d_RCI/0CS_ - d_ACTH/OCS_* where *d* refers to the treatment effect and ACTH refers to tetracosactide or CCMC for the respective networks evaluated. Nodes are weighted according to the number of included trials.

The network plot for each comparison–RCI versus tetracosactide (N=485 pooled patients)^6,14,27,29^ and RCI versus CCMC (N=183 pooled patients)^27–30^ is provided in Figure 2. The number of patients on RCI or synthetic ACTH treatments by outcomes are presented in Table 2.

**Table 2.** Distribution of Patients by Outcomes in the Included Studies

**Study**	**Treatment**	**Cessation of Spasms**	**Resolution of Hypsarrhythmia**	**Cessation of Spasms and Resolution of Hypsarrhythmia**
Baram et al, 1996^27^	RCI	93.3% (14/15)	86.7% (13/15)	86.7% (13/15)
Hrachovy et al, 1983^29^	RCI	75.0% (9/12)	75.0% (9/12)	75.0% (9/12)
O'Callaghan et al, 2017^6^	tetracosactide	70.5% (79/112)	68.5% (76/111)	68.5% (76/111)
Lux et al, 2004^14^	tetracosactide	76.0% (19/25)	88.9% (16/18)	76.0% (19/25)
Gowda et al, 2019^28^	CCMC	73.3% (11/15)	73.3% (11/15)	73.3% (11/15)
Wanigasinghe et al, 2015^30^	CCMC	36.7% (18/49)	18.4% (9/49)	18.4% (9/49)

Abbreviations: CCMC, corticotropin carboxymethyl-cellulose; RCI, repository corticotropin injection

The characteristics of clinical trials included in ITC are provided in Table 3. The clinical trials were published between 1983 and 2018. Patient selection criteria and patient age were similar for all clinical trials. The mean age ranged from 6.3 to 11.9 months. The RCI dose varied among the trials; however, there are no significant differences in efficacy with different doses.^31^ The dose of synthetic ACTH treatments was the same across trials in their respective networks. In all included trials, RCI or tetracosactide was better than oral corticosteroids in improving efficacy outcomes irrespective of oral corticosteroid dose. Studies included reported short-term response rates varying from two to four weeks.

**Table 3.** Characteristics of Clinical Trials on RCI or Synthetic ACTH Versus Oral Corticosteroids Included in the Indirect Treatment Comparison

**Author**	**Follow-up Assessment**	**Selection Criteria**	**Patient (N, age)**	**RCI or Synthetic ACTH Dose**	**Key Findings**	**Primary Outcome Assessed**
**Trials Evaluating Natural ACTH_1-39_ (RCI, Acthar^®^ Gel)**						
Baram et al, 1996^27^ (US; single center)	2 weeks; 4-24-hour video EEG for response.	Symptomatic or cryptogenic IS No prior steroid or ACTH treatment. Had hypsarrhythmia or its variants and epileptic myoclonic events.	N=29 2 to 21 mos (mean=6.3 mos)	150 units/m^2^/day in 2 divided doses	RCI was superior to oral corticosteroid treatment	Cessation of spasms and resolution of hypsarrhythmia
Hrachovy et al, 1983^29^ (US; single center)^a^	2 weeks; 24-hour video EEG for response.	IS and hypsarrhythmic EEG patterns. No prior ACTH or corticosteroid therapy.	N=24 3.5 to 24 mos (mean=8.2 mos)	20 units/day for 2 weeks and increased to 30 U/day	A higher proportion of patients responded to RCI than oral corticosteroid treatment	Cessation of spasms and resolution of hypsarrhythmia
**Trials evaluating synthetic ACTH_1-24_ (Tetracosactide, Synacthen^®^)**						
O'Callaghan et al, 2017^6^ (UK, Australia, Germany, New Zealand, Switzerland; multicenter)^b^	4 weeks; No mention of EEG duration. Absence of spasms for a 4-week period.	Clinical diagnosis of IS and hypsarrhythmic (or similar) EEG, ≤7 days before enrollment. No previous treatment for IS, including hormonal treatments and vigabatrin.	N=377 2 to 14 mos	0.5 mg [40 IU] on alternate days for 2 weeks, increased to 0.75 mg [60 IU] on alternate days after 1 week	A higher proportion of patients responded to tetracosactide than oral corticosteroid treatment	Cessation of spasms
Lux et al, 2004^14^ (UK; multicenter)	2 weeks; No mention of EEG duration. Absence of spasms for a 48-hour period.	Clinical diagnosis of IS and a hypsarrhythmic (or similar) EEG with almost continuous, high-voltage multifocal spike and wave. Both hypsarrhythmia and non-hypsarrhythmia patients included.	N=107 2 to 12 mos	0.5 mg [40 IU] on alternate days for 2 weeks, increased to 0.75 mg [60 IU] on alternate days after 1 week	A higher proportion of patients responded to tetracosactide than oral corticosteroid treatment	Cessation of spasms
**Trials Evaluating Synthetic ACTH_1-39_ (Corticotropin Carboxymethyl-cellulose, Acton Prolongatum^®^)**						
Gowda et al, 2019^28^ (India; single center)	4 weeks; No mention of EEG duration. Absence of spasms for a 48-hour period.	Diagnosis of West Syndrome (based on West Adelphi Group). No prior steroid use. Children with symptomatic, idiopathic, and cryptogenic etiologies.	N=34 2 to 60 mos (mean: 11.9 mos)	100 units per body surface area daily for 2 weeks	A higher proportion of patients responded to CCMC than oral corticosteroid treatment	Cessation of spasms
Wanigasinghe et al, 2015^30^ (Sri Lanka; single center)	2 weeks; 30-minute sleep EEG. Absence of spasms for a 48-hour period.	Newly diagnosed IS occurring in clusters, confirmed based on direct observation or in video telemetry. Only those with hypsarrhythmia included.	N=97 2 to 30 mos (mean=9.1 mos)	40 to 60 IU/every other day	CCMC was not superior to oral corticosteroid treatment	Cessation of spasms and resolution of hypsarrhythmia

Abbreviations: ACTH, adrenocorticotropic hormone; CCMC, corticotropin carboxymethyl-cellulose; EEG, electroencephalogram; IS, infantile spasms; mos, months; RCI, repository corticotropin injection; UK, United Kingdom; US, United States

^a^The study used a crossover design, where the data on outcomes were extracted including the crossover.

^b^Some patients received concomitant vigabatrin with prednisone or synthetic ACTH and the subjects were not randomly allocated to synthetic ACTH or prednisone.

### Risk of Bias Assessment

All included trials were of moderate quality, based on the Jadad score (Table S3).^20^ Four clinical trials described the randomization methodology. Only one trial reported using the double-blind method but did not describe the method of double-blind. In the Cochrane risk of bias assessment,^21^ all included trials had a low-to-medium risk of bias.^32^ The risk of bias related to blinding of outcome assessment was unclear for all trials. All had a low risk of bias in the incomplete outcome data and selective reporting domains. The results varied for the blinding of participants and personnel domain, with most trials reporting a high risk of bias and only a few trials reporting a low risk of bias. Risk of bias assessment was used as a tool to assess the strength of evidence of the individual studies based on the study design. The studies were not excluded based on the risk of bias assessment.

### Indirect Treatment Comparison

Given the small number of studies and inadequate sample size, the analysis included all selected studies for each respective network. Based on the model fit diagnostics, the fixed-effects model was used. Further, subgroup analyses were not conducted given the small number of studies in each network and inadequate sample size.

**Figure 3.**
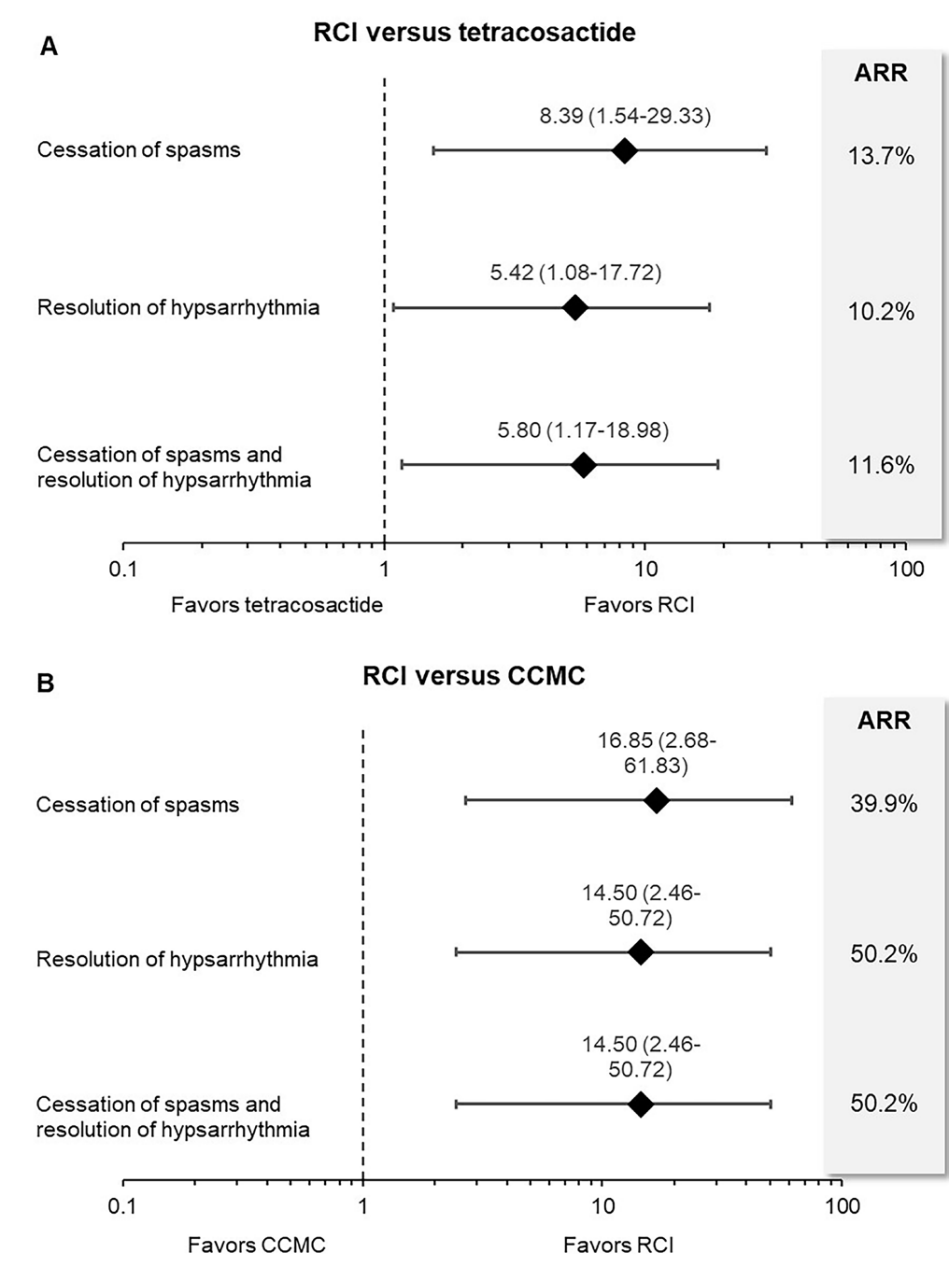
Forest Plots of Indirect Treatment Comparisons Between RCI and Synthetic ACTH Therapies Abbreviations: ACTH, adrenocorticotropic hormone; ARR, absolute risk reduction; RCI, repository corticotropin injection Results are reported as odds ratios (95% CrI). Odds Ratio >1 represents a favorable outcome for RCI. Findings are statistically not significant if the credible interval (CrI) includes 1.

RCI significantly improved cessation of spasms (OR = 8.39, 95% CrI = 1.54 to 29.33), resolution of hypsarrhythmia (OR = 5.42, 95% CrI = 1.08 to 17.72), and paired efficacy outcome (OR = 5.80, 95% CrI = 1.17 to 18.98) compared to tetracosactide (Figure 3A). RCI reduced the risk of spasms and/or hypsarrhythmia by 10.2% to 13.7% compared to tetracosactide. The risk reduction translates to an adjusted number needed to treat of four patients for the cessation of spasms and paired outcomes and five patients for the resolution of hypsarrhythmia. This suggests that for every four to five patients treated, RCI improved efficacy outcomes in one additional patient compared to synthetic tetracosactide.

Compared to CCMC, RCI had 16.9 times higher odds of achieving the cessation of spasms and 14.5 times higher odds of resolution of hypsarrhythmia and paired efficacy outcomes (Figure 3B). RCI reduced the risk of spasms and/or hypsarrhythmia by 39.9% to 50.2% compared to CCMC. The risk reduction for RCI versus CCMC translates to an adjusted number needed to treat of two patients for all efficacy outcomes.

## DISCUSSION

To our knowledge, this is the first network meta-analysis to compare the efficacy of RCI to synthetic ACTH treatments. Based on the limited evidence, findings from the indirect comparison suggest that RCI may be a better treatment option for improving the cessation of spasms and/or resolution of hypsarrhythmia relative to synthetic ACTH therapies.

Given the lack of direct evidence, the results of this study add to the emerging literature on the efficacy of RCI and provide new comparative evidence that lays the basis for future prospective studies. Effective treatments for infantile spasms should result in complete control of spasms and resolution of hypsarrhythmia;^2^ clinical examination focuses on identifying the underlying condition, while EEG can provide certainty in diagnosis. The ITC examined outcomes based on both clinical and EEG criteria to comprehensively evaluate RCI and synthetic ACTH therapies. The present analysis is limited to available clinical outcomes.

The findings should be interpreted in the context of the limitations. It should be noted that the data on RCI and synthetic ACTH therapies are limited and inconsistent in study design, outcomes assessment, and treatment dose based on the clinical recommendations at the time the trial was conducted. The risk of bias varied across the studies. The risk of bias assessment tool was used to assess the strength of the body of evidence of the individual studies included in the analysis. Infantile spasms is a rare disease with limited published clinical trials. These limited clinical trials may present some bias based on the study design; however, they meet the criteria for applicability, study population, and reporting of outcome measures. The lead time to treatment also varied across the studies that may result in some differences in the efficacy of these treatments. For example, the studies on CCMC were conducted in Asian countries that have limited resources and thus had longer lead times to treatment. Further, the findings from this review demonstrated a lack of head-to-head randomized controlled trials comparing RCI and synthetic ACTH treatments and inconsistencies in study design for the evidence base. Additionally, the evidence base from the included clinical trials was dated, using recommended dosing strategies at the time each of these trials was conducted. Randomized controlled trials are considered the gold standard study design for guiding clinical decision-making; however, due to the lack of direct evidence, an ITC of limited clinical trials was conducted to compare the efficacy of RCI and synthetic ACTH treatments.

The clinical trials included in the analysis used variable dosing of the treatments, consistent with the clinical recommendations available at the time the trial was conducted.^7,14^ It is anticipated that RCI would remain efficacious when compared to synthetic ACTH therapies accounting for dose variations in RCI and oral corticosteroids. The efficacy of low-dose RCI (20 to 30 IU/day) was similar to high-dose RCI (150 units/m^2^/day; approximately 60 IU/day) in a dose-comparison trial.^31^ Oral corticosteroid dose in RCI clinical trials was 2 mg/kg/day (approximately 20 mg/day), whereas oral corticosteroid dose for synthetic ACTH trials ranged from 40 to 60 mg/day. Similarly, the variations in oral corticosteroid dose are unlikely to affect the findings. First, there is limited evidence from well-controlled trials and low-quality evidence from observational studies supporting the efficacy of an oral corticosteroid dose-response relationship for the treatment of infantile spasms.^33^ Second, in all included trials, RCI or tetracosactide was better than oral corticosteroids in improving efficacy outcomes irrespective of oral corticosteroid dose. Further, a large prospective study suggests improved effectiveness of RCI compared to high-dose oral corticosteroids.^7^ Finally, the Bayesian approach determines the true treatment effect by abstracting the treatment effect of the control arm, reducing the bias emanating from any dose-dependent response of oral corticosteroids.^34^ The cross-trial differences between RCI and synthetic ACTH resulting from corticosteroid dose variations were accounted for by measuring the treatment effect relative to the common arm in these trials.

Other limitations are inherent to the meta-analysis methodology. The results represent the statistical aggregation of data from the trials included in the network and depend on the quality and comparability of its included trials. Thus, the results should be consistent but may not be completely identical to those of individual clinical trials. Despite an extensive literature search, only four clinical trials were included for each RCI versus synthetic ACTH comparison. The limited number of trials in each network made it challenging to adequately assess heterogeneity. The wide credible intervals may be attributable to a small number of trials and a limited sample size. Finally, the disease etiology was not reported consistently across trials, therefore, there is a possibility of bias from potential treatment effect modifiers. The systematic literature review was limited to studies published in English.

The study findings shed light on the differences in the efficacy of RCI over synthetic ACTH therapies. Direct head-to-head trials are needed to further confirm the findings. Further trials are needed with a larger number of participants, robust methodology, standardized and valid outcome measures, and detailed reporting, including other underlying etiology, adverse events, long-term neurodevelopmental effects, time to response, duration of response, and mortality. Since infantile spasms is a relatively rare disorder, recruitment of large numbers of patients into randomized controlled trials may be achieved through continuing multi-center collaboration. Future research needs to evaluate other efficacy outcomes, including the time to and duration of cessation of spasms, as clinically meaningful endpoints. Longer follow-up studies may help capture patients who experience recurrence of spasms.

## CONCLUSIONS

These results provide insights into the comparative evidence on ACTH therapies and provide a blueprint to inform the design of future prospective studies in this area. The study findings suggest that RCI may be an effective treatment option for infantile spasms. Additional comparative studies will provide a better understanding of the relative effectiveness of RCI against other treatments for infantile spasms. Overall, the treatment preference should be driven by patient preferences, drug availability, efficacy, adverse effects, and clinical recommendations.

### DISCLOSURE OF CONFLICTS OF INTEREST

This study was sponsored by Mallinckrodt Pharmaceuticals, Inc. Repository corticotropin injection (RCI; Acthar^®^ Gel) is a product of Mallinckrodt Pharmaceuticals.

Dr. Duchowny and Dr. Devine are research collaborators in this study and have no disclosures. Dr. Niewoehner and Dr. Wan are employees of Mallinckrodt Pharmaceuticals. Dr. Chopra was an independent research consultant paid by Mallinckrodt Pharmaceuticals.

### AUTHOR CONTRIBUTIONS

BD, GJW, IC, JN, and MSD were involved in study conceptualization and design. BD and IC were involved in the statistical analysis design and conducting analysis. GJW, JN, and IC were involved in acquisition of the data. BD, GJW, and IC contributed to the interpretation of the data. GJW and IC were involved in the drafting of the manuscript. BD, GJW, IC, JN, and MSD provided critical review and revision of the manuscript for important intellectual content. BD, GJW, IC, JN, and MSD provided the final approval of the version to be published.

All authors had full access to all of the data in this study. All named authors meet the International Committee of Medical Journal Editors (ICJME) criteria for authorship for this article, take responsibility for the integrity of the work as a whole, and have given approval for this version to be published. All authors have reviewed and approved the final draft of the manuscript, have obtained the required ethical approvals, have given necessary attention to ensure the integrity of the work, and have agreed to bear the applicable publication charges if their manuscript is accepted for publication.

## Supplementary Material

Supplementary Material
