# Supplementary material for: A Systematic Literature Review and Indirect Treatment Comparison of Efficacy of Repository Corticotropin Injection versus Synthetic Adrenocorticotropic Hormone for Infantile Spasms
Source: J Health Econ Outcomes Res. 2021 Jan 27;8(1):1–9. doi: 10.36469/jheor.2021.18727 (PMC7839629; doi:10.36469/jheor.2021.18727)
Supplement: Supplementary Material [file jheor_2021_8_1_18727_50533.pdf]

Journal of Health Economics and Outcomes Research

Supplementary Online Material

Duchowny MS, Chopra I, Niewoehner J, Wan GJ, Devine B. A systematic literature review and indirect treatment comparison of efficacy of repository corticotropin injection versus synthetic adrenocorticotropic hormone for infantile spasms. *JHEOR*. 2021;8(1):1-9
doi:10.36469/jheor.2021.18727

Table S1. Search Strategy for MEDLINE via PubMed (executed September 30, 2020)
Table S2. Overview of Studies Evaluating RCI and Synthetic ACTH Therapies Excluded from the Meta-analysis
Table S3. Jadad Score and Cochrane Risk of Bias Assessment of Included Trials

This supplementary material has been provided by the authors to give readers additional information about their work.

This is an open-access article distributed under the terms of the Creative Commons Attribution 4.0 International License (CCBY-4.0). View this license's legal deed at http://creativecommons.org/licenses/by/4.0 and legal code at http://creativecommons.org/licenses/by/4.0/legalcode for more information.

Table S1. Search Strategy for MEDLINE via PubMed (executed September 30, 2020)

Line no.	Search term
1	"Spasms, Infantile"[MeSH] OR "salaam spasm*"
2	"Adrenocorticotropic Hormone"[MeSH] OR "Cosyntropin"[MeSH]
3	"Acthar" OR "corticotropin*"
4	#1 AND (#2 OR #3)
5	Filters:<br>• Language: English<br>• Species: Humans<br>Study design: Clinical Trial; Meta-Analysis; Pragmatic Clinical Trial; Randomized Controlled Trial; Systematic Reviews; Journal Article

Table S2. Overview of Studies Evaluating RCI and Synthetic ACTH Therapies Excluded from the Meta-analysis

Author, Year	Study Design	Population	Treatments (Regimen)	Key Findings	Reason for Exclusion
**Studies Evaluating RCI**					
Dressler et al, 2015[1] (Austria)	Prospective, Randomized cross-over, Single center	Cases with a confirmed diagnosis of West Syndrome based on EEG monitoring; no prior treatment; age=0-27 months	RCI: 150 IU/m^2, tapering over 28 days Keto diet	Cessation of spasms in 54% on RCI compared to 45% on keto diet. No significant difference	Clinical trial study with no common comparator arm for comparison with synthetic ACTH treatment; RCI compared to keto diet
Knupp et al, 2016[2] (US)	Prospective, Multicenter	New onset of IS; age = 2-24 months	RCI: 150 IU/m^2/d, tapering to 10 IU/m^2/d OCS: 40 mg/d, tapering to 10 mg/d	55% infants on RCI showed clinical remission and resolution of hypsarrhythmia compared to 39% on OCS (P<0.001)	Observational prospective study design
Hrachovy et al, 1994[3] (US)	RCT, Single center	Newly diagnosed cases of IS with demonstrated hypsarrhythmia (EEG findings), cryptogenic or symptomatic group, not previously been treated with ACTH or OCS	High dose RCI: 150 U/m^2/d Low dose RCI: 20 to 30 U/d	No significant difference between response rate, defined as cessation of spasms and disappearance of hypsarrhythmia (high dose: 50%, low dose: 58%)	Dose comparison clinical trial study
Snead et al, 1989[4] (US)	Prospective, Single center	Diagnosis of IS, made by clinical history and EEG; mean age = 43.3 months	RCI: 75 - 150 IU/m^2/d	Cessation of spasms and resolution of hypsarrhythmia in 14 out of 15 children; 93.3% response	Observational prospective study design
Dreifuss et al, 1986[5] (US)	RCT, Multicenter	IS documented by a hypsarrhythmic or modified hypsarrhythmic pattern (EEG findings), no prior use of treatment with ACTH, OCS, or nitrazepam; mean age: 8.4 months	RCI: 40 IU/d Nitrazepam: 0.2 mg/kg/d in two or 1 mg twice daily	75-100% reduction in spasm frequency was 57% for ACTH and 52% for nitrazepam. Both groups showed significant reduction in spasms from baseline. No significant difference between groups.	Clinical trial study with no common comparator arm for comparison with synthetic ACTH treatment; RCI compared to nitrazepam

Table S2. Overview of Studies Evaluating RCI and Synthetic ACTH Therapies Excluded from the Meta-analysis

Author, Year	Study Design	Population	Treatments (Regimen)	Key Findings	Reason for Exclusion
Lombroso et al, 1983[6] (US)	Prospective, Single center	Diagnosis of typical or atypical IS, presence of EEGs with hypsarrhythmia, presence of cryptogenic or symptomatic cases; age: <24 months	RCI: 110 units/m^2/d OCS: 2 mg/kg/d	48% and 43% patients on RCI achieved cessation of spasms and resolution of hypsarrhythmia compared to 38% and 35% on OCS, respectively	Observational prospective study design
Snead et al, 1983[7] (US)	Prospective, Single center	Diagnosis of IS or other types of myoclonic seizures, or those with intractable seizures; mean age = 6.2 to 47.8 months	RCI: 75 - 150 IU/m^2/d OCS: 3 mg/kg/d	Seizure control ranged from 67%-100% of the patients on RCI vs. 0%-59% on OCS. Resolution of hypsarrhythmia in 29/30 patients (96.7%) compared to 11/22 patients on OCS (50%)	Observational prospective study design
Singer, 1980[8] (US)	Retrospective chart review, Single center	Seizure disorder characterized by massive myoclonic jerks, extensor spasms or a combination of the two, and an EEG pattern of hypsarrhythmia; modal age: 5 months	RCI: 80 IU every other day	Cessation of spasms in 74.5% with 5 to 6 months of RCI therapy (27/31 in early treatment group and 14/24 in late treatment group)	Single arm and observational retrospective study design
**Studies Evaluating Synthetic ACTH$_{1-24}$ (Tetracosactide, Synacthen®)**					
Jones et al, 2015[9] (Canada)	Retrospective case series review, Single center	Cases of newly diagnosed IS with demonstrated clinical spasms and hypsarrhythmia, cryptogenic or symptomatic IS; median age: 4 months	Tetracosactide: 1.9 mg/m^2 tapering to 0.06 mg/m^2 OCS: 4.5 mg/kg/d tapering to 1.5 mg/kg/d	Cessation of spasms and resolution of hypsarrhythmia in 80% children on synthetic ACTH$_{1-24}$ vs. 20% on OCS	Observational retrospective study design
Ibrahim et al, 2010[10] (Pakistan)	Retrospective chart review, Single center	Diagnosed with IS, hypsarrhythmia on EEG and no diagnosis of tuberous sclerosis; no prior experience with vigabatrin, ACTH; mean age: 6.5 months	Tetracosactide: 40-80 IU daily Vigabatrin: 12.5–150 mg/kg/d	No statistically significant difference observed between 2 treatments (ACTH: 9/18 [50%]; vigabatrin: 21/38 [55.3%])	Observational retrospective study design with no common comparator arm for comparison with RCI
Cohen-Sadan et al, 2009[11] (Israel)	Retrospective chart review, Multicenter	Patients with idiopathic West Syndrome; age: 2 to 9 months	Tetracosactide: 100 IU; alternate days Vigabatrin: 100–180 mg/kg/d	Cessation of spasms and resolution of hypsarrhythmia in 87% patients on synthetic ACTH$_{1-24}$ compared to 78% on vigabatrin	Observational retrospective study design with no common comparator arm for comparison with RCI
Lin et al, 2006[12] (Taiwan)	Prospective, Multicenter	Diagnosis of West Syndrome or IS with hypsarrhythmia on EEG, never treated with ACTH or OCS; mean age: 8.2 months	Tetracosactide: 2.5 IU daily and tapered	46/53 (86.8%) had cessation of spasms, of which 35/46 (76.1%) had complete cessation of spasms	Single arm with observational prospective study design

Table S2. Overview of Studies Evaluating RCI and Synthetic ACTH Therapies Excluded from the Meta-analysis

Author, Year	Study Design	Population	Treatments (Regimen)	Key Findings	Reason for Exclusion
Azam et al, 2005[13] (Pakistan)	Retrospective chart review, Single center	Characteristic seizures of IS, hypsarrhythmia on EEG, no previous experience with ACTH or OCS; mean age: 11 months	Tetracosactide: 20-40 IU OCS: 2-3 mg/kg/d	No significant difference between treatment groups: ACTH: 27/33 (82%) responded (11/33 [33.3%] remained spasms free); OCS: 51/72 (71%) responded (17/72 [24%] remained spasm-free)	Observational retrospective study design
Kurokawa et al, 1980[14] (Japan)	Cross-sectional survey, Multicenter	Children with West Syndrome or Lennox-Gastaut syndrome characterized by spasms starting in infancy; age: ≤6 years	Tetracosactide: 10-20 mg/d OCS: 10 mg/kg/d	Cessation of spasms in 75.6% patients on synthetic $ACTH_{1-24}$ 38.0% on OCS. Resolution of hypsarrhythmia in 33.5% of patients on synthetic $ACTH_{1-24}$ vs. 20.4% on OCS.	Observational cross-sectional study design
**Studies Evaluating Synthetic $ACTH_{1-39}$ (Corticotropin Carboxymethyl-cellulose, Acton Prolongatum)**					
Angappan et al, 2019[15] (India)	RCT, Single center	Newly diagnosed cases of West syndrome confirmed by clinical assessment and EEG, did not receive previous treatments with ACTH, OCS or zonisamide; median age: 8-10 years	Corticotropin carboxymethyl-cellulose: 30 IU/d to a maximum daily dose of 60 IU/d Zonisamide: 4-8 mg/kg/day to a maximum daily dosage of 25 mg/kg/day	Cessation of epileptic spasms was observed in 27% vs. 40% of patients on zonisamide and ACTH1-39, respectively. Resolution of hypsarrhythmia at 6 weeks was 36% vs. 71% for those on zonisamide and ACTH1-39, respectively. No significant difference between groups was observed.	Clinical trial study with no common comparator arm for comparison with synthetic RCI; Corticotropin carboxymethyl-cellulose compared to zonisamide

Abbreviations: ACTH, adrenocorticotropic hormone; EEG, electroencephalogram; IS, infantile spasms; IU, international units; OCS, oral corticosteroids; RCI, repository corticotropin injection; RCT, randomized controlled trial; US, United States

Table S3. Jadad Score and Cochrane Risk of Bias Assessment of Included Trials

Cochrane Risk of Bias Assessment

Author, Year	Random Sequence Generation (Selection Bias)	Allocation Concealment (Selection Bias)	Blinding of Participants and Personnel (Performance Bias)	Blinding of Outcome Assessment (Detection Bias)	Incomplete Outcome Data (Attrition Bias)	Selective Reporting (Reporting Bias)
Baram et al, 1996[16]	Low	Unclear	Low, Personnel	Unclear	Low	Low
Gowda et al, 2018[17]	Low	Low	High	Unclear	Low	Low
Hrachovy et al, 1983[18]	Low	Low	Low	Unclear	Low	Low
Lux et al, 2004[19]	Low	Low	High	Unclear	Low	Low
O'Callaghan et al, 2017[20]	High	Low	High	Unclear	Low	Low
Wanigasinghe et al, 2015[21]	Low	Low	High	Unclear	Low	Low

Jadad Score

Author, Year	Randomization	Method of Randomization	Double-blind	Method of Double-blind	Withdrawals/ Dropouts	Total Score
Baram et al, 1996[16]	1	1	0	0	1	3
Gowda et al, 2018[17]	1	1	0	0	1	3
Hrachovy et al, 1983[18]	1	0	1	0	1	3
Lux et al, 2004[19]	1	1	0	0	1	3
O'Callaghan et al, 2017[20]	0	0	0	0	1	1
Wanigasinghe et al, 2015[21]	1	1	0	0	1	3

Supplementary Materials References

1. Dressler A, Trimmel-Schwahofer P, Reithofer E, et al. The ketogenic diet versus ACTH in the treatment of infantile spasms: A prospective randomised study. In: 11th European Paediatric Neurology Society Congress. Vienna, Austria: European Journal of Paediatric Neurology; 2015.
2. Knupp KG, Coryell J, Nickels KC, et al. Pediatric Epilepsy Research C. Response to treatment in a prospective national infantile spasms cohort. *Ann Neurol.* 2016;79:475-484. doi:10.1002/ana.24594
3. Hrachovy RA, Frost JD, Jr., Glaze DG. High-dose, long-duration versus low-dose, short-duration corticotropin therapy for infantile spasms. *J Pediatr.* 1994;124:803-806. doi:10.1016/s0022-3476(05)81379-4
4. Snead OC, 3rd, Benton JW, Jr., Hosey LC, et al. Treatment of infantile spasms with high-dose ACTH: Efficacy and plasma levels of ACTH and cortisol. *Neurology.* 1989;39:1027-1031. doi:10.1212/wnl.39.8.1027
5. Dreifuss F, Farwell J, Holmes G, Joseph C, Lockman L, Madsen JA, Minarcik CJ, Jr., Rothner AD, Shewmon DA. Infantile spasms. Comparative trial of nitrazepam and corticotropin. *Arch Neurol.* 1986;43:1107-1110. doi:10.1001/archneur.1986.00520110007005
6. Lombroso CT. A prospective study of infantile spasms: clinical and therapeutic correlations. *Epilepsia.* 1983;24:135-158. doi: 10.1111/j.1528-1157.1983.tb04874.x.
7. Snead OC, 3rd, Benton JW, Myers GJ. ACTH and prednisone in childhood seizure disorders. *Neurology.* 1983;33:966-970. doi:10.1212/wnl.33.8.966
8. Singer WD, Rabe EF, Haller JS. The effect of ACTH therapy upon infantile spasms. *J Pediatr.* 1980;96:485-489. doi:10.1016/s0022-3476(80)80706-2
9. Jones K, Snead OC, 3rd, Boyd J, Go C. Adrenocorticotropic hormone versus prednisolone in the treatment of infantile spasms post vigabatrin failure. *J Child Neurol.* 2015;30:595-600. doi:10.1177/0883073814533148
10. Ibrahim S, Gulab S, Ishaque S, Saleem T. Clinical profile and treatment of infantile spasms using vigabatrin and ACTH--a developing country perspective. *BMC Pediatr* 2010;10:1. doi:10.1186/1471-2431-10-1
11. Cohen-Sadan S, Kramer U, Ben-Zeev B, et al. Multicenter long-term follow-up of children with idiopathic West syndrome: ACTH versus vigabatrin. *Eur J Neurol.* 2009;16:482-7. doi:10.1111/j.1468-1331.2008.02498.x
12. Lin HC, Young C, Wang PJ, Lee WT, Shen YZ. ACTH therapy for Taiwanese children with West syndrome -- efficacy and impact on long-term prognosis. *Brain Dev.* 2006;28:196-201. doi:10.1016/j.braindev.2005.07.002
13. Azam M, Bhatti N, Krishin J. Use of ACTH and prednisolone in infantile spasms: Experience from a developing country. *Seizure.* 2005;14:552-556. doi:10.1016/j.seizure.2005.08.009.

14. Kurokawa T, Goya N, Fukuyama Y, Suzuki M, Seki T, Ohtahara S. West syndrome and Lennox-Gastaut syndrome: A survey of natural history. *Pediatrics.* 1980;65:81-88.

15. Angappan D, Sahu JK, Malhi P, Singhi P. Safety, tolerability, and effectiveness of oral zonisamide therapy in comparison with intramuscular adrenocorticotropic hormone therapy in infants with West syndrome. *Eur J Paediatr Neurol.* 2019;23:136-142. doi:10.1016/j.ejpn.2018.09.006

16. Baram TZ, Mitchell WG, Tournay A, Snead OC, Hanson RA, Horton EJ. High-dose corticotropin (ACTH) versus prednisone for infantile spasms: A prospective, randomized, blinded study. *Pediatrics.* 1996;97:375-379.

17. Gowda VK, Narayanaswamy V, Shivappa SK, Benakappa N, Benakappa A. Corticotrophin-ACTH in comparison to Prednisolone in West Syndrome - A randomized study. Indian J Pediatr. 2019;86:165-170. doi:10.1007/s12098-018-2782-1

18. Hrachovy RA, Frost JD, Jr., Kellaway P, Zion TE. Double-blind study of ACTH vs prednisone therapy in infantile spasms. *J Pediatr.* 1983;103:641-645. doi:10.1016/s0022-3476(83)80606-4

19. Lux AL, Edwards SW, Hancock E, et al. The United Kingdom Infantile Spasms Study comparing vigabatrin with prednisolone or tetracosactide at 14 days: A multicentre, randomised controlled trial. *Lancet.* 2004;364:1773-1778. doi:10.1016/S0140-6736(04)17400-X

20. O'Callaghan FJ, Edwards SW, Alber FD, et al. Safety and effectiveness of hormonal treatment versus hormonal treatment with vigabatrin for infantile spasms (ICISS): A randomised, multicentre, open-label trial. *Lancet Neurol.* 2017;16:33-42. doi:10.1016/S1474-4422(16)30294-0

21. Wanigasinghe J, Arambepola C, Sri Ranganathan S, Sumanasena S, Attanapola G. Randomized, single-blind, parallel clinical trial on efficacy of Oral Prednisolone versus Intramuscular Corticotropin on immediate and continued spasm control in West Syndrome. Pediatr Neurol. 2015;53:193-199. doi:10.1016/j.pediatrneurol.2015.05.004
